# Oxidative Stress in COPD

**DOI:** 10.3390/jcm8111953

**Published:** 2019-11-13

**Authors:** María Magallón, María Mercedes Navarro-García, Francisco Dasí

**Affiliations:** 1 Instituto de Investigación Sanitaria INCLIVA, Fundación Investigación Hospital Clínico Universitario Valencia, Avda. Menéndez y Pelayo, 4, 46010 Valencia, Spain; mariamagallon94@gmail.com (M.M.); mer_navarro2002@yahoo.es (M.M.N.-G.); 2 Rare Respiratory Diseases Group, Department of Physiology, School of Medicine, University of Valencia, Avda. Blasco Ibáñez, 17, 46010 Valencia, Spain

**Keywords:** oxidative stress, COPD, alpha-1 antitrypsin deficiency, antioxidants

## Abstract

Numerous studies over the years have shown that oxidative stress plays a major role in the development of the disease. Oxidative stress involvement in COPD opens up the possibility of using antioxidant therapies in the treatment of the disease. However, so far, these therapies have shown no clinical benefit indicating that more basic research efforts are needed to understand the underlying mechanisms by which oxidative stress leads to the development of COPD.

Chronic obstructive pulmonary disease (COPD) is a chronic progressive lung disease characterised by progressive and non-reversible airway obstruction with systemic manifestations and major comorbidities affecting patients’ quality of life [1]. COPD is currently the fourth most common cause of death worldwide and expected to be the third by 2030 if preventive measures are not taken [2]. Cigarette smoking (CS) is by far the most common cause of COPD [3]. The World Health Organization estimates that up to 73% of COPD deaths in high-income countries and over 90% in developing countries are related to tobacco smoking. Other factors such as environmental pollution, exposure to biomass smoke and genetic factors have been reported to be involved in the development of the disease [4].

Disease onset occurs most often in people over 40 years of age and elderly populations that have a personal history of smoking. However, although about 90% of COPD are smokers, only 15–20% of chronic smokers (rising to 50% in heavy smokers) will develop clinically relevant COPD so it is extremely important to identify who is at highest risk and why. In addition, current therapies for COPD are largely ineffective and the development of new treatments for the disease have been hindered because the basic mechanisms that lead to COPD initiation and progression are poorly understood [2,3].

The pathophysiology of COPD is associated with obstruction of the smaller airways and tissue remodeling that occurs as a consequence of chronic inflammation and destruction of the lung parenchyma in response to various environmental (CS among others) and genetic stimuli. Despite public information campaigns by governments and medical societies to prevent and quit smoking, one-fifth of the population continues to smoke, and in each puff, 10^15^ oxidants and more than 4000 chemical compounds are inhaled [5]. Numerous evidences over the years have shown that oxidative stress, a condition in which the amount of oxidizing molecules overwhelms cellular antioxidant defences, and the associated oxidative damage to biomolecules located in the cells of the airways play an important role in the pathogenesis of COPD [5,6]. Importantly, oxidative stress and oxidative damage persist in the airways of COPD patients after smoking cessation [7]. The sources of oxidizing substances responsible for the development of oxidative stress are numerous and varied and include oxidants derived from environmental pollution (nanoparticles from industries, car exhaust fumes, etc.) and cigarette smoke and endogenous substances released by leukocytes and macrophages involved in the chronic inflammation process observed in the lungs of COPD patients [3]. These endogenous substances include reactive oxygen species (ROS) and reactive nitrogen species (RNS) which are capable on the one hand of causing oxidative damage to biomolecules hindering normal cell function and on the other hand of activating the expression of redox-sensitive pro-inflammatory transcription factors such as NF-κB to maintain a chronic pro-inflammatory state that increases the production of cell-derived ROS such as NADPH oxidase, the xanthine/xanthine oxidase system and the heme-peroxidases which have been found to be elevated in COPD patients [3]. Similarly, nitric oxide (a RNS) is capable of reacting with superoxide anion to generate peroxynitrite, a powerful oxidizing agent exhibiting a wide array of tissue damaging [8,9]. On the other hand, CS also affects the antioxidant defences of the lung by irreversibly damaging glutathione (GSH) and decreasing the activity of transcription factors such as the nuclear factor-erythroid 2-related factor 2 (*Nrf2*), a transcription factor that under conditions of oxidative stress initiates the transcription of antioxidant genes and their proteins [5,10].

Similarly, oxidative stress is involved in the pathogenesis of alpha-1 antitrypsin deficiency (“the genetic COPD”), a hereditary condition characterised by low plasma levels of alpha-1 antitrypsin, that increases the risk of COPD and liver disease in these patients [11]. Our research group has shown increased levels of hydrogen peroxide and low glutathione and diminished catalase activity that lead to oxidative damage of lipids, proteins and DNA that could be involved in the lung damage observed in some patients with the disease [12,13].

A recent review by Mc Guinness and Sapey in the Journal of Clinical Medicine focuses on the role of oxidative stress in the pathogenesis of COPD [2]. The authors emphasize the central role of oxidative stress in COPD onset and progression which opens up a rationale for the use of antioxidant therapies (either targeting oxidative stress or enhancing endogenous antioxidant defences) in the treatment of the disease. However, as indicated by the authors, these therapies have not shown (so far) any clinical benefit for COPD patients suggesting that more efforts in COPD basic research are needed to improve our knowledge of the underlying biology and metabolic changes elicited by oxidative stress in airways epithelial cells that will help us identify which patients are at higher risk of developing COPD and to develop novel and more effective antioxidant therapies.
